# Acknowledgement to Reviewers of *Micromachines* in 2016

**DOI:** 10.3390/mi8010019

**Published:** 2017-01-11

**Authors:** Micromachines Editorial Office

**Affiliations:** MDPI AG, St. Alban-Anlage 66, 4052 Basel, Switzerland; micromachines@mdpi.com

The editors of *Micromachines* would like to express their sincere gratitude to the following reviewers for assessing manuscripts in 2016.

We greatly appreciate the contribution of expert reviewers, which is crucial to the journal’s editorial process. We aim to recognize reviewer contributions through several mechanisms, of which the annual publication of reviewer names is one. Reviewers receive a voucher entitling them to a discount on their next MDPI publication and can download a certificate of recognition directly from our submission system. Additionally, reviewers can sign up to the service Publons (https://publons.com) to receive recognition. Of course, in these initiatives we are careful not to compromise reviewer confidentiality. Many reviewers see their work as a voluntary and often unseen part of their role as researchers. We are grateful to the time reviewers donate to our journals and the contribution they make.

If you are interested in becoming a reviewer for *Micromachines*, see the link at the bottom of the webpage http://www.mdpi.com/reviewers.

The following reviewed for *Micromachines* in 2016:
Abadie, J.Hong, Chien-ChongPatel, SaurinAbate, AdamHong, SukjoonPaust, NilsAbdel-Motaleb, Ibrahim M.Hoorfar, MinaPawashe, ChytraAbdolvand, RezaHornak, Larry A.Pawashe, ChytraAbelmann, LeonHorwat, DavidPetsev, Dimiter N.Abidi, KhalidHsu, Jyh-PingPhan, Hoang-PhuongAbidian, Mohammad RezaHsu, Shan-huiPhillips, Scott T.Aditya, SheelHu, GuoqingPierron, Olivier N.Afkhami, ShahriarHu, NingPiraino, FrancescoAgrawal, NitinHuang, HuiPister, Kristofer S.J.Ahmad, ZeeshanHuang, LiangliangPoenar, Daniel P.Ahn, Sung-HoonHuang, Ngan F.Polimeno, AntoninoAi, YeHuang, WeiminPollet, JeroenAkhter, MahbubHuang, YanyiPrecup, Radu-EmilAlan, TuncayHuang, ZhaorongPrickril, BenAlexeev, IlyaHughes, Michael P.Pu, Suan HuiAlexis, FrankHwang, DavidPuchades, IvanAmin, HayderHwang, YonghaQasaimeh, MohammadAmstad, EstherIkonomidou, Vasiliki N.Qi, ZenanAoyagi, TakahiroIliescu, CiprianQian, ShizhiAphale, Sumeet S.Ino, KosukeQian, YouArima, ValentinaIovenitti, PioQuack, NielsAstana, RajivIyer, Subramanian S.Qubeissi, Mansour AlAtkinson, Gary M.Jacobi, IanQue, LongAusländer, SimonJambovane, SachinQuintela, AntonioBahadur, VaibhavJanssen, Kris Pieter FransRabenorosoa, KantyBai, JingJavanmard, MehdiRakotondrabe, MickyBandyopadhyay, DipankarJensen, Brian D.Ramanujan, Raju V.Banerjee, AshisJeon, JaeseokRaptis, IoannisBanerjee, DebjyotiJeon, Tae-JoonRebhan, BernhardBanks, CraigJeong, Ok ChanReboud, JulienBarbero, SergioJia, YuRehberg, IngoBareket, LilachJiao, LihongReinhardt, AlexandreBarillaro, GiuseppeJiao, ZhenjunRembe, ChristianBarrett, JohnJimenez, MelanieRen, CarolynBarrios, Carlos AnguloJin, QinghuiRen, KangningBasabe-Desmonts, LourdesJoe, Daniel J.Ren, YongBasarab, AdrianJones, MatthewRen, YukunBasu, AnirbanJones, Thomas B.Renzoni, FerruccioBatabyal, SubrataJoye, Colin D.Rhee, MinsoungBausells, JoanJuang, Yi-JeRipka, PavelBelfiore, Nicola PioJun, Seong ChanRivadeneyra, AlmudenaBenazouz, AymenJurado-Sánchez, BeatrizRoth, GunterBensaoula, AbdelhakJwo, Dah-JingRuther, PatrickBen-Yakar, AdelaKaajakari, VilleRyu, Seok ChangBerg-Sorensen, KirstineKalantar Zadeh, KouroshSaakes, MichelBergstrom, PaulKamiński, MarcinSabatini, AngeloBertacco, RiccardoKaneta, TakashiSakar, Mahmut SelmanBerthier, JeanKang, Dong-KuSales, SalvadorBevly, DavidKang, Sung HoonSallese, Jean-MichelBeyne, EricKang, Tae GonSalter, PatrickBhattarai, KeshavKang, YuejunSalvan, GeorgetaBiswal, Sibani LisaKaradimitriou, NikolaosSan, HaishengBityurin, NikitaKarimi, HassanSantiago-Aviles, Jorge J.Blaikie, RichardKaushik, AjeetSarioglu, A. FatihBleser, GabrieleKawahara, TomohiroSato, KaeBludov, YuKeating, AdrianScaini, DenisBourouina, TarikKeplinger, FranzSchaefer, Hans-EckhardtBreedveld, PaulKevin, Ge QiSchenato, LucaBrouzes, EricKhoshmanesh, KhashayarSchmid, AndreasBrunet, PhilippeKiani, AmirkianooshSchmid, SilvanBuma, TakashiKieninger, JochenSchmidt, MichaelBunch, Joseph ScottKim, CheolGiSchmidt, Oliver G.Butler, Jason E.Kim, Deok-HoSchneider, Ben H.Cabral, João T.Kim, DonghyunSchwemmer, FrankCai, ZiliangKim, Dong-KwonScotti, GianmarioCaponetti, LauraKim, HanseupSelleri, StefanoCarnevale, MauroKim, Hee ChanSelvaganapathy, RaviCass, ThonyKim, Hyun JungSeo, Tae SeokCastrejon-Pita, Alfonso A.Kim, JoonwonSerra, Christophe A.Cetin, BarbarosKim, JungkyuSesé, JavierCeyssens, FrederikKim, KeekyoungShapiro, BenjaminChaganti, Srinivasa ReddyKim, KyumanSheinin, AntonChan, Leanne Lai-HangKim, Min JunSheta, BassemChan, Yung-KuanKim, Myun-SikShih, SteveChang, Chih-WeiKim, SeokShih, Wen-PinChang, Suk TaiKim, Sun MinShin, SehyunCharmet, JeromeKim, Yong TaeShoffstall, Andrew J.Chen, Chao Yu PeterKinahan, David JShu, Yi-ChungChen, ChengpengKiwa, ToshihikoSibbett, ScottChen, Hung-YiKlingbeil, LasseSing, Swee LeongChen, JunningKoblischka, Michael RudolfSmith, Henry I.Chen, Kuan-NengKoltay, PeterSmits, EdsgerChen, Yu-BinKong, Siu-KaiSolvas, Xavier Casadevall IChen, Yung-YuKoo, ChiwanSong, Yong-HaCheney, Marcos A.Kosa, GaborSong, YongxinCheng, Chao-MinKosmrlj, AndrejSooryakumar, RatnasinghamCheng, Ching-HsiangKozai, Takashi D. Y.Spira, Micha E.Cheng, MarkKraft, UlrikeSrigrarom, SutthiphongCheng, XuanhongKrause, Hans-JoachimSteeneken, PeterCheng, YaKrause, SteffiSteenson, David PaulChiang, Kai-WeiKrull, Ulrich J.Stelzle, MartinChiao, Jung-ChihKulkarni, SantoshStoychev, GeorgiChin, Ken K.Kumar, AlokeSu, Quang T.Chiolerio, AlessandroKumar, SandeepSun, Chia-LiangChitnis, GirishKwok, Chee YeeSun, JiashuCho, Hyoung JinKymissis, IoannisSun, MengCho, Seong YunLai, Chao SungSundaram, Kalpathy B.Cho, YounghakLake, Robert ASung, JaeyongChoi, DanielLal, AmitSwami, NathanChoi, HongsooLam, Raymond H. W.Syms, RichardChoi, SeokheunLambert, PierreTabib-Azar, MassoodChoi, SungyoungLamprou, DimitriosTakamatsu, HiroshiChowdhury, SagarLancaster, RobertTaklo, Maaike M. V.Chowdhury, SazzadurLang, WalterTam, HwayawChristensen, Kenneth A.Lee, Byeong HaTan, Cher MingChu, VirginiaLee, Chung-HoonTan, Chuan SengChun, HongguLee, DonghwaTan, Say HwaChung, AramLee, JoshuaTanaka, YoChung, Jae-HyunLee, JungwooTang, FeiChung, Wen-YawLee, Seok JaeTang, Shi-YangCiofani, GianniLee, Sung SikTang, William CCitterio, DanielLee, SunwooTao, KaiClavica, FrancescoLee, SunwooTaylor, SteveCliffel, David E.Lee, Won GuTemiz, YukselCochran, SandyLee, WonheeThalhammer, GregorCohen, NettaLelievre, SophieThickett, Stuart C.Collins, David J.Lenci, StefanoThielemann, ChristianeComi, ClaudiaLi, BinThompson, Carl V.Cong, YongzhengLi, Cheuk-WingThorsen, ToddConnelly, DanielLi, Paul C.H.Thuo, MartinCooke, MichaelLi, PengTichem, MarcelCopic, DavorLi, XiuJun JamesTixier-Mita, AgnesCorigliano, AlbertoLi, XuToan, Nguyen VanCosta, ManuelLi, YangminTowe, EliasCosta, MariaLiang, XinTowfighian, ShahrzadCrane, NathanLim, Kian-MengTravagliati, MarcoCranford, StevenLima, RuiTremblay, CharlesCrowe, JohnLin, Chih-LangTrouillon, RaphaëlCulbertson, Christopher T.Lin, Chih-MingTsao, Chia-WenCulmer, PeteLin, Chih-mingTsuchiya, ToshiyukiCummins, GerardLin, JonqlanTummala, Rao R.Czilwik, GregorLin, M.-T.Tung, Yi-ChungDe Marco, CarmelaLin, Yow-JonTurner, Kevin T.De Volder, MichaelLink, WolfgangUzunlar, ErdalDean, RobertLinzon, YoavVai, Mang I.Deiss, FrédériqueLiu, BifengVan Dam, R. MichaelDelville, Jean-PierreLiu, Cheng-HsienVan Ness, KirkDen Toonder, JaapLiu, DonVelev, OrlinDeutsch, MordechaiLiu, Jefferson Z.Vellekoop, Michiel J.Di Carlo, DinoLiu, TingtingVerkouteren, R. MichaelDi Cioccio, LéaLiu, XinyuVettore, AntonioDi Natale, CorradoLiu, ZhenyuVietzorreck, LarissaDickerson, AndrewLo, RogerVillard, CatherineDickey, MichaelLo, Yu-hwaVinegoni, ClaudioDidar, TohidLoskill, PeterVisconte, CarmenDidar, TohidLu, JianVolland, BurkhardDietzel, AndreasLu, Jun WeiVorobiev, AndreiDijksman, J. FritsLu, XinyuVrana, Nihal EnginDikin, Dmitriy A.Lucia, RomanoWadsak, WolfgangDimaki, MariaLucyszyn, StepanWalton, JeffreyDimov, StefanLuo, QuantianWang, ChengDing, XiaoyunLuo, XiaolongWang, GuirenDonaldson, NickLuo, YanhuaWang, GuoanDrazer, GermanLuttge, ReginaWang, MoranDual, JurgMa, YuWang, PengtaoDuarte Campos, Daniela FilipaMacka, MirekWang, SihongDutoit, BertrandMadrenas, JordiWANG, YixinDzung, DaoMahjoubi, HoseinWang, ZhenfengEaton, ShaneMalamud, DanielWang, ZhenfengElbuken, CaglarMandracci, PietroWang, ZuankaiEllis, JonthanMani, VigneshwaranWare, TaylorEmadi, ArezooManjare, ManojWarkiani, Majid EbrahimiEndo, TatsuroMao, LeidongWarren, RoseanneFair, Richard B.Marcos, MarcosWasserman, DanielFaivre, DamienMariani, StefanoWatanabe, WataruFan, ShermanMarkos, ChristosWathuthanthri, IshanFan, Shih-KangMarta, Ruiz LlataWebster, JohnFearing, RonaldMartinez, AndresWei, GuowuFeldman, MartinMartinez-Duarte, RodrigoWei, JianjunFelton, Samuel MMartins-Lopes, PaulaWei, QingshanFernández Romero, J. M.Maruthamuthu, VenkatWeigand, AnnikaFernandino, MariaMastrangeli, MassimoWeinberger, Leor SFerraro, DavideMastrangelo, CarlosWhite, HenryFerraro, PietroMathews, ScottWhite, Robert D.Fischer, GeorgMatsko, AndreyWiegerink, RemcoFischlechner, MartinMatson, Eric T.Wikswo, JohnFouillet, YvesMatsuo, ShigekiWikswo, JohnFourati, NajlaMcCain, Megan L.Willis, PeterFournel, FrankMcKendry, JonathanWindmill, JamesFrançais, OlivierMcnamara, ShamusWirth, Christopher LFranke, ThomasMeagher, RobertWiśniowski, PiotrFricke, KatjaMelling, DanielWong, Kam SingFriedman, GaryMéndez, A.Wood, DavidFriedt, Jean-MichelMendis, RajindWu, JayneFu, Lung-MingMeng, XuXi, Heng-DongFU, TaotaoMerlo, SabinaXie, HuikaiFüchslin, RudolfMichael, AronXie, HuikaiFurutani, K.Micic, MiodragXu, JiawenGagnon, Zachary R.Millet, Larry J.Xu, JieGangloff, DorianMin, Jung-joonXu, ShijieGao, AnmingMinea, Alina AdrianaXuan, XiangchunGao, YandongMistura, GiampaoloXue, WeiGarg, AkhilMiyashita, ShuheiYaish, Yuval E.Gauthier, MichaëlMohd Yasin, FaisalYan, JizeGhayesh, MergenMoon, Chan-wooYang, Eui-HyeokGhayesh, Mergen H.Moriceau, HubertYang, MengsuGhisi, AldoMorley, Nicola AYang, MoGibson, DesMortazawi, AmirYang, Ruey-JenGlenni, CraigMosadegh, BobakYang, ShengyuanGlynn, Macdara T.Mossi, KarlaYang, ShikuanGogolides, EvangelosMuguruma, HitoshiYang, YiGoldhawk, DonnaMulloni, VivianaYang, ZhenchuanGomez, FrankMumtaz, KamranYasukawa, TomoyukiGomez, Frank A.Murphy, Coleen T.Yeh, Li-HsienGopinath, JulietMuthuswamy, JitYellen, Benjamin B.Gracias, DavidMyers, OliverYeo, JunyeobGrant, Peter M.Nabki, FrédéricYeom, JunghoonGrbovic, DragoslavNelson, BradleyYi, Yun-BoGreener, JesseNg, AlphonsusYin, HuabingGueorguiev, G.K.Ng, Sum HuanYook, Jong-GwanGuijt, Rosanne M.Ngamson, BongkotYu, Chin-pingGulati, ShellyNikumb, SuwasYu, CunjiangGupta, BhagwatiNisato, GiovanniYu, Hua-ZhongHagelauer, AmelieNisisako, TakasiYu, Xiao-YingHall, NealNiu, XizeYuan, JunqiHan, ArumNoels, LudovicYuan, LeiHancock, Matthew J.Nugen, SamYun, YeoheungHane, KazuhiroO’Mahony, ConorZaghloul, MonaHao, GuangboOakey, JohnZahn, Jeffrey D.Hao, QiangOberoi, SnehaZangle, ThomasHarazim, Stefan M.Oda, MasahiroZara, Jason M.Harper, JasonOesterschulze, EgbertZarzar, Lauren D.Hashemi, NastaranOh, Sang SoonZemp, Roger J.Hashimoto, ShigehiroOhodnicki, Paul R.Zervantonakis, IoannisHe, XiminOk, JongZhai, LeiHegemann, DirkOldham, KennZhan, HaifeiHelseth, Lars EgilOliver, NickZhang, HongboHenry, M. DavidOrobtchouk, RegisZhang, JunfengHerrera-May, AgustinOrtolani, MicheleZhang, QijinHesketh, PeterOu, HaiyanZhang, XiaoyuHess, Dennis W.Ouyang, MengxingZhang, XumingHickman, James J.Ozevin, DidemZhang, XunliHigo, AkioPaeng, Dong-GukZhao, HongHilber, WolfgangPagnotta, LeonardoZhao, XinHildreth, OwenPalego, CristianoZhe, JiangHiller, KarlaPanat, Rahul PadmakarZheng, BoHippler, RainerPancrazio, JosephZheng, YiHirai, YoshikazuPandey, SantoshZheng, YingHirtz, MichaelPang, WeiZhou, GuangyaHitt, Darren L.Pannasch, SebastianZhou, JianHjort, KlasParedes, JacoboZhou, WanluHo, Ho PuiPark, ChangsooZhou, YunshenHo, Tsung-YiPark, ChoongbaeZhu, LikunHöer, Jörg J.Park, Je-KyunZhu, YongHoettges, KaiPark, Joong YullZimmerman, WIlliamHollis, Ralph L.Park, JoontaekZivkovic, Vladimir

